# The effect of hemodialysis on ocular changes in patients with the end-stage renal disease

**DOI:** 10.1080/0886022X.2019.1635494

**Published:** 2019-07-04

**Authors:** Guijiang Sun, Rui Hao, Longli Zhang, Xueying Shi, Kaiwen Hei, Lijie Dong, Fang Wei, Aili Jiang, Bo Li, Xiaorong Li, Yifeng Ke

**Affiliations:** a Department of Kidney Disease and Blood Purification Treatment, Tianjin Institute of Urology, The Second Hospital of Tianjin Medical University, Tianjin, China;; b Tianjin Eye Hospital, Tianjin Key Laboratory of Ophthalmology and Vision Science, Nankai University Eye Hospital, Clinical College of Ophthalmology, Tianjin Medical University, Tianjin, China;; c Tianjin Medical University Eye Hospital, Tianjin Medical University Eye Institute, Tianjin, China

**Keywords:** End stage kidney disease, hemodialysis, subfoveal choroidal thickness, retinal arteriolar caliber, retinal venular caliber

## Abstract

**Background:** Numerous metabolic parameters can be changed during hemodialysis in the end-stage renal disease (ESRD) caused by systemic diseases, such as diabetes mellitus, hypertension. Some ocular parameters also can be variable due to the changes after hemodialysis. This study evaluates the effects of ocular parameters, including best-corrected visual acuity (BCVA), intraocular pressure (IOP), central macular thickness (CMT), subfoveal choroidal thickness (SFCT), retinal arteriolar caliber (RAC), retinal venular calibre (RVC), in ESRD patients following hemodialysis.

**Materials and methods:** Two-hundred and two ESRD patients were recruited resulting in 404 eyes evaluations. All patients underwent hemodialysis in the Dialysis Unit of the Second Hospital of Tianjin Medical University. BCVA, CMT, IOP, SFCT, RAC and RVC were evaluated before and after hemodialysis. Systemic parameters were collected such as age, body weight, systolic blood pressure (SBP), diastolic blood pressure (DBP), duration of hemodialysis, body weight changes, high density lipoprotein cholesterol (HDLC), low density lipoprotein cholesterol (LDLC), very low density lipoprotein cholesterol (VLDLC), glycosylated hemoglobin (HbA1c).

**Results:** The causes of ESRD patients included chronic glomerulonephritis (*n* = 65), diabetes mellitus (*n* = 60), hypertensive nephrosclerosis (*n* = 37), and other causes (*n* = 40). In our study, BCVA (*p* = .817), CMT (*p* = .252) and IOP (*p* = .978) did not significantly change after hemodialysis. SFCT significantly decreased from 254.29 ± 69.36 μm to 235.54 ± 659.90 μm (*p* = .002) following hemodialysis. SFCT changes were significantly correlated with SBP (*p* = .042) and body weight changes (*p* = .044). The RAC and RVC were dilated significantly (*p* = .033, *p* = .007). RVC changes were correlated with baseline DBP (*p* = .003), HDLC (*p* = .009), LDLC (*p* = .004) and changes in DBP (*p* = .037) and body weight (*p* = .001).

**Conclusion:** Hemodialysis can affect various ocular parameters including SFCT, RAC and RVC, which changed significantly following hemodialysis. Whereas BCVA, IOP and CMT did not change after hemodialysis in ESRD patients. The systemic compensatory mechanisms of the changes in SBP, DBP, body weight following hemodialysis need further study.

## Introduction

Renal disease, particularly the end-stage renal disease (ESRD), is a costly and disabling condition with a high mortality rate [1]. Patients with ESRD are generally treated using a blood-filtration mechanism, such as hemodialysis. During this treatment, numerous metabolic parameters can be changed, especially blood urea, sodium, potassium and blood glucose levels. These fluctuations result in changes in blood vessels and extracellular fluids [2]. Meanwhile, some ocular parameters can also be variable due to these changes in ESRD patients. However, the changes of ocular parameters vary. It has been reported that best corrected visual acuity (BCVA) improves after a single hemodialysis session [3]. Some research indicated central macular thickness (CMT) remain unchanged [2] or even decreased [4] after hemodialysis. Other study have reported that subfoveal choroidal thickness (SFCT) decreased after a single hemodialysis [5]. Additionally, various studies have shown IOP to increase [6], decrease [2] or remain unchanged [7]. It also reported that retinal arteriole caliber did not change, but retinal venules dilated after hemodialysis [8]. Moreover, most of the reports were small-scale study and few reports had compared ocular parameter changes with hemodialysis in different causes of ESRD.

Since the effects of hemodialysis on ocular parameters remain unclear, our study examines a large population with the short-term changes in ophthalmologic findings before and after a single hemodialysis session. The relationships between these changes and systemic parameters (e.g., body weight, SBP and DBP,) are evaluated. Furthermore, the effects of ocular parameters are evaluated and compared in patients with different causes of ESRD.

## Methods

This prospective, cross-sectional study included 404 eyes of 202 patients with stage 5 ESRD who underwent maintenance hemodialysis in the Second Hospital of Tianjin Medical University from September 2014 to July 2017. Informed consent was obtained from all subjects, and the study conducted adhered to the Declaration of Helsinki. Institutional review board approval was also obtained from the Second Hospital of Tianjin Medical University.

All the patients in this study underwent three 4-h hemodialysis sessions each week for at least 3 months. The patients, who were less than 18-year-old and more than 75-year-old, had pregnancy, malignant tumor, infective disease, immunological disease, were eliminated. The patients with ocular disease that would interfere with ocular examination and measurement, such as macular degeneration, vitreous hemorrhage, ocular trauma and any other ocular surgery, laser therapy or intraocular injection within 3 months of enrollment were excluded. The patients with smoking habit were also excluded.

Fresenius 4008 s dialysis machine (Fresenius Medical Care AG & Co. KGaA, Bad Homburg, Germany) was used with Polysulfone membrane dialyzer and low molecular weight heparin as an anticoagulant. The bicarbonate in bipolar reverse osmosis water used with a dialysate flow rate of 500 mL/min and blood flow rate of 200–300 mL/min. Predialytic and post-dialytic body weight were measured in each patient. SBP and DBP were measured during hemodialysis. Other systemic parameters were recorded such as age, HbA1c, duration of hemodialysis, HDLC, LDLC, VLDLC and blood glucose.

All the patients underwent a detailed ophthalmologic examination before and after hemodialysis. BCVA was obtained using retinoscopy optometry and IOP was measured by using tonometer (Tonopachy NT-530P, Nidek Co., LTD., Tokyo, Japan), CMT and SFCT were administrated using RTVue SD-OCT system (RTVue-XR 100 Avanti software v.6.1, Optovue, Inc., Fremont, CA, USA). Fundus photograph was obtained using a digital non-mydriatic retinal camera (CR-DGi; Canon, Tokyo, Japan).

Retinal arteriolar caliber (RAC) and retinal venular caliber (RVC) were measured in terms of retinal fundus photographs. These photographs were taken using a digital non-mydriatic retinal camera after dilating the pupils with 1% tropicamide. Two retinal images of each eye were obtained, one centered on the optic disc and another centered on fovea. The analysis of image used a computer-based program, Interactive Vessel Analysis software (IVAN) program (University of Wisconsin, US), to measure retinal vascular caliber [9]. Retinal vascular caliber was measured through a specified zone of 1 disc diameter away from the optic disc margin. Based on the revised Knudtson-Parr-Hubbard formula, retinal arteriolar and venular calibers were summarized as RAC and RVC, respectively [10]. Reproducibility of retinal vascular measurements was high, with intra-grader intraclass correlation coefficients [95% confidence interval] 0.98 (0.97–0.99) for RAC and 0.94 (0.92–0.96) for RVC.

### Statistical analysis

Collected outcomes were analyzed using SPSS v.17.0 for Windows (SPSS, Inc., Chicago, IL, USA). The normality of the distribution of data was determined using the Kolmogorov–Smirnov test. All continuous data were normally distributed. Descriptive statistics were presented as mean ± SD. For continuous data, the ocular parameters before and after dialysis were compared with Student’s paired *t*-test. Pearson’s correlation coefficient test was utilized to compare the correlation of ocular parameters and systemic parameters in the hemodialysis period. A result was considered significant if the *p* values were <.05.

## Results

A total of 202 patients (109 male and 93 female patients) were enrolled, resulting in 404 eye examinations. The general and systemic parameters are summarized in Table 1. Average age of all patients was 54.76 ± 11.10 years and subjects had been undergoing maintenance hemodialysis for a mean period of 75.24 ± 66.04 months. Mean HbA1c in hemodialysis patients combined with DM was 7.33%±1.30%. The mean body weight was 66.00 ± 11.98 kg. The average SBP and DBP in hemodialysis patients were 140.54 ± 24.93 mmHg and 80.84 ± 14.67 mmHg, respectively. The mean HDLC, LDLC and VLDLC in hemodialysis patients were 0.86 ± 0.30 mmol/L, 2.37 ± 0.71 mmol/L and 0.80 ± 0.41 mmol/L, respectively. The causes of ESRD patients are summarized in Table 2, These causes include chronic glomerulonephritis (*n* = 65), diabetes (*n* = 60), hypertensive nephrosclerosis (*n* = 37) and other causes (*n* = 40).

**Table 1.** The general and sysmetic parameters of 202 patients before HD.

	Mean ± SD	Range
Age (years)	54.76 ± 11.10	26–72
HbA1c (%)	7.33 ± 1.30	5.5–10.2
Duration of HD (months)	75.24 ± 66.04	4–485
Body weight (kg)	66.00 ± 11.98	40.2–100.4
Body mass index (kg/m^2^)	23.77 ± 3.52	16.35–33.54
SBP (mmHg)	140.54 ± 24.93	76–194
DBP (mmHg)	80.84 ± 14.67	48–114
HDLC (mmol/L)	0.86 ± 0.30	0.31–1.81
LDLC (mmol/L)	2.37 ± 0.71	1.16–4.37
VLDLC (mmol/L)	0.80 ± 0.41	0.16–1.91
Blood glucose (mmol/L)	5.82 + 2.52	3.27–20.77

HbA1c: glycosylated hemoglobin; SBP: Systolic blood pressure; DBP: Diastolic blood pressure; HDLC: high density lipoprotein cholesterol; LDLC: low density lipoprotein cholesterol; VLDLC: very low density lipoprotein cholesterol; HD: hemodialysis.

**Table 2.** The causes for ESRD in 202 hemodialysis patients.

Disease	*n*	%
CGN	65	25.79
DM	60	23.81
Hypertension	37	14.68
PKD	9	3.57
Lupus nephritis	7	2.78
RC in nephrectomized patient	4	1.59
CIN	6	2.38
Oxalosis	5	1.98
FSG	2	0.79
Unknown	7	2.78
Total	202	100

CGN: chronic glomerulonephritis; DM: Diabetes Mellitus; PKD: polycystic kidney disease; RC: Renal carcinoma; CIN: chronic interstitial nephritis; FSG: focal segmental glomerulonephritis.

For systemic changes, the mean body weight decreased significantly from 66.00 ± 11.98 kg to 63.15 ± 9.42 kg after hemodialysis (*p* < .001). SBP and DBP also decreased significantly following the hemodialysis; the differences were 20.13 ± 16.06 mmHg and 12.57 ± 11.87 mmHg, respectively (*p* < .001). Systemic findings are shown in Table 3.

**Table 3.** Systemic findings before and after hemodialysis treatment.

	Pre-HD	Post-HD	Changes	*p* Value
Body weight (kg)	66.00 ± 11.98	63.15 ± 9.42	3.12 ± 1.07	<.001
SBP (mmHg)	140.54 ± 24.93	118.49 ± 21.11	20.13 ± 16.06	<.001
DBP (mmHg)	80.84 ± 14.67	67.41 ± 12.63	12.57 ± 11.87	<.001

SBP: Systolic blood pressure; DBP: Diastolic blood pressure.

For ophthalmic findings, in general, tthe mean SFCT decreased significantly from 254.29 ± 69.36 μm to 235.54 ± 659.90 μm (*p* = .002) after hemodialysis. The RAC and RVC dilated remarkably from 139.37 ± 33.43 μm, 219.15 ± 49.46 μm to 145.71 ±  38.95 μm, 231.73 ± 43.43 μm (*p* = .033, *p* = .007), respectively. The SFCT, RAC and RVC had significant changes in all patients group, chronic glomerulonephritis group, diabetes mellitus group, hypertension group and other causes group following the hemodialysis except for the RVC in other causes group (*p* = .132). There were no significant difference in BCVA, IOP and CMT in all patients group, chronic glomerulonephritis group, hypertension group and other causes group after hemodialysis. While the BCVA and CMT were significantly changed in diabetes mellitus group following the hemodialysis (*p* = .042, *p* = .04, respectively) (Table 4).

**Table 4.** Effect of hemodialysis on ocular changes.

	Pre-HD	Post-HD	*p* Value
All patients			
BCVA (LogMAR)	0.65 ± 0.32	0.66 ± 0.31	.817
IOP (mmHg)	14.17 ± 3.16	14.95 ± 2.74	.978
CMT (*μ*m)	255.72 ± 35.29	258.19 ± 48.26	.252
SFCT (*μ*m)	254.29 ± 69.36	235.54 ± 59.90	.002
RAC (*μ*m)	139.37 ± 33.43	145.71 ± 38.95	.033
RVC (*μ*m)	219.15 ± 49.46	233.73 ± 43.43	.007
Chronic glomerulonephritis			
BCVA (LogMAR)	0.59 ± 0.35	0.62 ± 0.44	.458
IOP (mmHg)	16.33 ± 3.18	15.98 ± 3.05	.229
CMT (*μ*m)	237.58 ± 46.24	243.12 ± 51.12	.322
SFCT (*μ*m)	244.29 ± 51.34	228.54 ± 53.59	.01
RAC (*μ*m)	133.37 ± 40.43	142.71 ± 46.95	.02
RVC (*μ*m)	213.75 ± 42.76	227.23 ± 46.89	.02
Diabetes Mellitus			
BCVA (LogMAR)	0.72 ± 0.57	0.78 ± 0.46	.042^a^
IOP (mmHg)	12.32 ± 2.39	12.96 ± 1.85	.371
CMT (*μ*m)	298.07 ± 57.77	280.15 ± 60.12	.040^a^
SFCT (*μ*m)	277.31 ± 67.38	253.24 ± 71.06	.001
RAC (*μ*m)	142.12 ± 25.45	150.36 ± 29.17	.03
RVC (*μ*m)	228.57 ± 46.82	245.42 ± 44.20	.005
Hypertension			
BCVA (LogMAR)	0.61 ± 0.36	0.59 ± 0.29	.394
IOP (mmHg)	15.53 ± 2.18	15.48 ± 2.05	.316
CMT (*μ*m)	250.18 ± 41.24	254.82 ± 32.62	.614
SFCT (*μ*m)	253.29 ± 71.53	230.54 ± 74.62	.001
RAC (*μ*m)	133.53 ± 23.42	142.16 ± 27.52	.02
RVC (*μ*m)	211.17 ± 55.82	221.42 ± 60.18	.01
Other causes			
BCVA (LogMAR)	0.50 ± 0.26	0.56 ± 0.38	.214
IOP (mmHg)	13.83 ± 2.59	13.68 ± 3.05	.646
CMT (*μ*m)	245.58 ± 33.24	248.12 ± 32.12	.532
SFCT (*μ*m)	250.29 ± 61.34	233.54 ± 73.59	.02
RAC (*μ*m)	141.53 ± 23.42	146.16 ± 27.52	.11
RVC (*μ*m)	222.57 ± 55.82	235.42 ± 50.20	.015

a BCVA and CMT have significant difference after HD in Diabetes Mellitus patients.

BCVA: best-corrected vision acuity; IOP: intraocular pressure; CMT: central macular thickness; SFCT: subfoveal choroid thickness; RAC: retinal arteriolar caliber; RVC: retinal venular caliber; HD: hemodialysis.

An analysis was performed to determine if hemodialysis-induced changes in SFCT, RVC, RAC were correlated with baseline age, duration of hemodialysis, body weight, ultrafiltration rate, blood glucose, HDLC, LDLC, VLDLC and the change in SBP, DBP and body weight. The results indicated that changes in SFCT positively correlated with a body weight change (*p* = .044) and baseline SBP (*p* = .042) (Table 5). RAC changes correlated negatively with all systemic parameters (Table 6). While the changes in RVC were markedly correlated with baseline DBP (*p* = .003), HDLC (*p* = .009), LDLC (*p* = .004), DBP change (*p* = .037) and body weight change (*p* = .001) (Table 7).

**Table 5.** Determinants of subfoveal choroidal thickness changes.

Factors	Subfoveal choroidal thickness change		
	Correlation	95% CI (lower, upper)	*p* Value
Age	0.069	(−0.246, 0.250)	.59
Hemodialysis duration	−0.078	(−0.308, 0.212)	.543
Body weight (kg)	−0.167	(−0.381, 0.048)	.192
Baseline SBP	−0.243	(−0.506, −0.01)	.042^a^
Baseline DBP	−0.211	(−0.466, 0.098)	.097
Body weight change	−0.255	(−0.457,−0.017)	.044^a^
SBP change	−0.117	(−0.256, −0.11)	.215
DBP change	0.167	(0.032, 0.293)	.127
Ultrafiltration rate	−0.035	(−0.076, 0.54)	.544
HDLC (mmol/L)	0.047	(−0.249, 0.276)	.715
LDLC (mmol/L)	0.103	(−0.164, 0.404)	.423
VLDLC (mmol/L)	0.049	(−0229, 0.322)	.704
Blood glucose (mmol/L)	−0.21	(−0.363, 0.018)	.097

a Positive correlation with subfoveal choroidal thickness.

SBP: Systolic blood pressure; DBP: Diastolic blood pressure; HDLC: high-density lipoprotein cholesterol; LDLC: low-density lipoprotein cholesterol; VLDLC: very low-density lipoprotein cholesterol.

**Table 6.** Determinants of central retinal arteriolar caliber changes.

Factors	Retinal arteriolar caliber change		
	Correlation	95% CI (lower, upper)	*p* Value
Age	−0.191	(−0.353, 0.121)	.134
Hemodialysis duration	0.158	(−0.094, 0.400)	.217
Body weight (kg)	0.093	(−0.186, 0.335)	.468
Baseline SBP	0.233	(0.02, 0.555)	.066
Baseline DBP	0.137	(−0.158, 0.314)	.283
Body weight change	−0.055	(−0.320,−0.189)	.67
SBP change	−0.213	(−0.406, 0.11)	.275
DBP change	0.297	(0.002, 0.543)	.587
Ultrafiltration rate	0.054	(−0.027, 0.114)	.714
HDLC (mmol/L)	0.027	(−0.234, 0.282)	.835
LDLC (mmol/L)	0.045	(−0.236, 0.329)	.727
VLDLC (mmol/L)	0.096	(−0.200, 0.400)	.452
Blood glucose (mmol/L)	0.231	(−0.005, 0.394)	.069

SBP: Systolic blood pressure; DBP: Diastolic blood pressure; HDLC: high-density lipoprotein cholesterol; LDLC: low-density lipoprotein cholesterol; VLDLC: very low-density lipoprotein cholesterol.

**Table 7.** Determinants of central retinal venular caliber changes.

Factors	Retinal venular caliber change		
	correlation	95% CI (lower, upper)	*p* Value
Age	−0.003	(−0.288, 0.492)	.984
Hemodialysis duration	0.388	(0.219, 0.605)	.182
Body weight (kg)	0.001	(−0.294, 0.248)	.994
Baseline SBP	−0.194	(−0.494, 0.111)	.128
Baseline DBP	0.366	(0.095, 0.578)	.003^a^
Body weight change	0.423	(0.234, 0.601)	.001^a^
SBP change	−0.133	(−0.396, 0.158)	.745
DBP change	0.087	(0.159, 0.009)	.037^a^
Ultrafiltration rate	−0.105	(−0.209, 0.94)	.394
HDL cholesterol (mmol/L)	−0.327	(0.072, 0.598)	.009^a^
LDL cholesterol (mmol/L)	−0.362	(−0.499,−0.115)	.004^a^
VLDL cholesterol (mmol/L)	0.032	(−0.317, 0.292)	.804
Blood glucose(mmol/L)	0.202	(−0.029, 0.377)	.112

a Positive correlation with retinal venular caliber.

SBP: Systolic blood pressure; DBP: Diastolic blood pressure; HDLC: high-density lipoprotein cholesterol; LDLC: low-density lipoprotein cholesterol; VLDLC: very low-density lipoprotein cholesterol.

## Discussion

The prevalence of ESRD is increasing due to the ascending incidence of risk disease for ESRD, such as diabetes, metabolic syndrome and hypertension [11]. The main etiology for ESRD is variable and controversial. In the recent study, Oh et al. reported glomerulonephritis; diabetic nephropathy; hypertensive nephropathy; polycystic kidney disease were the most common causative diseases in descending order [12]. While Chelala and Tow showed that diabetic nephropathy was the leading risk disease during the chronic kidney disease [2,8]. Our data indicated glomerulonephritis; diabetic nephropathy; hypertensive nephropathy were the top three risk diseases in the ESRD patients. We thought this difference may due to the ethnic difference and diet habbit during the process of ESRD. Caucasian preferred high caloric diet compared with Asian, leading to a high prevalence of diabetes and metabolic syndrome disease.

The choroid has a rich vascular network and highest blood supply per organ area. During a hemodialysis session, numerous metabolic parameters change. Ultrafiltration removes excess fluid from plasma, which leads to an increasing in the plasma protein concentration. Blood volume depletion is equilibrated by vascular refilling from the interstitial and intracellular space [13]. This may lead to the change in choroidal thickness (CT) following a hemodialysis session. Jung et al. studied on 28 eyes in 19 patients with ESRD and reported that SFCT increased following a hemodialysis session and was correlated with decline in SBP [14]. They proposed that the increase in SFCT might be associated with the choroidal autoregulatory control of ocular hemodynamics, shifting of fluid and molecules between the plasma and choroidal interstitium. However, There were conflicting results on the effects of SFCT following a hemodialysis session. Chang et al. recruited 31 patients resulting in 54 eyes and showed that choroidal thickness was reduced in all areas following a hemodialysis session. The reduction in choroidal thickness was correlated with body weight loss, serum osmolarity and SBP [15]. Ishibazawa et al. analyzed 41 patients with ESRD and found the SFCT decreased in all eyes after hemodialysis [16]. Chen and Çelikay also showed that choroidal thickness was decreased in the patients with ESRD following a hemodialysis session [5,17]. Our results indicated that the changes in SFCT were reduced remarkably following a hemodialysis session. The reduction in choroidal thickness was correlated with body weight loss and baseline SBP. According to our observation on these studies, only one paper showed SFCT was increased whereas all the other papers have demonstrated reducing SFCT after hemodialysis session. The difference between these conflicting results may be related to the methodology used to measure choroidal thickness and the demographic variation of the populations in different study groups [17]. In addition, the mechanism of the SFCT thinning may be the result of the increase in choroidal vascular and nonvascular smooth muscle contraction which was due to the activation of the sympathetic autonomic nervous system. It is well known that choroidal circulation was controlled primarily by the extrinsic autonomic system. Sympathetic activation triggered by blood volume depletion might cause choroidal vascular and nonvascular smooth muscle constriction [18], which led to a decrease in CT.

Another major finding of this study is that the hemodialysis results in dilatation of the retinal venules and arterioles. In present study, the RAC and RVC were significantly increasing after the hemodialysis. When all possible variables were taken into account, the RVC changes between pre and post dialysis were correlated with DBP, LDLC, HDLC and changes in body weight and DBP. Although the mean RAC rose significantly after the hemodialysis, the change in RAC did not correlate with any systemic parameters. This result was partly different from the previous study. Tow et al. analyzed on 24 patients and showed that the mean RVC increased significantly. After dialysis, the change in RVC was correlated only with the body weight change. However, the mean RAC did not change significantly after the hemodialysis [4]. We are of the opinion that the conflicting results of RAC were due to the size of the different population. The previous study had a small sample size compared with our study. We thought that might be the key factor for the different results.

The mechanism of the dialysis-associated vasodilatation is not clear. Usually, endothelium-dependent vasodilatation was thought to be the major vasodilatation mechanism in ESDR patients [19]. Endothelium-dependent dilatation in response to flow-mediated shear stress is impaired in the resistant vasculature from patients with ESRD and finally result in contraction of resistance vasculature due to blunted blood flow and the lack of nitric oxide (NO) contribution [20]. However, dilatation of the retinal microvasculature after dialysis is thought to occur secondary to the release of vasoactive factors, such as nitric oxide, nitrotyrosine, Asymmetric dimethylarginine (ADMA), heat shock proteins, in response to the reduced intravascular volume [21]. For example, asymmetric dimethylarginine, an inhibitor of nitric oxide synthase, accumulates in renal failure [22], The elevated levels of ADMA would decrease NO production. However, it was removed by dialysis, resulting in enhanced nitric oxide production and consequent vasodilatation. And so did the nitrotyrosine [20].

In our study, BCVA and CMT did not change remarkably following a single hemodialysis session except DM groups. BCVA and CMT changes were insignificantly correlated with any systemic parameters (data not show). This result is supported by Chelala’s study [2]. They analyzed on 49 eyes from 49 chronic kidney-disease patients and demonstrated that neither BCVA nor CMT significantly changed after the hemodialysis. Our study also revealed that the mean BCVA and CMT were reduced only in diabetic ESRD patients after the hemodialysis (Table 4). Diabetic patients were prone to visual disturbance such as decreasing VA due to retinopathy. These effects were more common in diabetic hemodialysis patients [23]. Nevertheless, the previous results of BCVA changes varied in diabetic hemodialysis patients. Ghasemi reported osmotic changes during hemodialysis, induced by hyperglycemia before the starting of insulin therapy, played an important role in lens overhydration by sorbitol accumulation [24]. Tai et al. studied groups of poorly controlled diabetic patients and reported that the alteration in refractive index of the lens was responsible for the refractive changes of the eye [25]. On the other hand, Wiemer et al. reported that no significant correlations were found between changes in blood glucose and refractive states of the cornea and lens in diabetic patients [26]. The BCVA reduction may due to a series of happenings caused by the hemodialysis microvascular changes, including ocular hypotension, decrease in zonular tension, shallowing of the anterior chamber, thickening of the lens and finally a myopic shift in refractive power of the eye [27]. There are also some other papers showing that BCVA reduction or loss after the hemodialysis was caused by the ischemic optic neuropathy [28,29]. In addition, decrease in CMT during hemodialysis has also been involved in the hemodialysis microvascular changes [30]. Each 50 μm changes in retinal thickness caused 0.15 D changes in refractive error, this may explain the decrease in CMT induced BCVA reduction during hemodialysis session [22].

The IOP change following hemodialysis remains controversial. Many conflicting IOP results have been published on the effects of hemodialysis. Çelikay et al. concluded that IOP did not significantly change after hemodialysis in ESRD patients [17]. Our study also supports the above-mentioned findings. However, Chelala et al. have demonstrated a slight but significant decrease in IOP with hemodialysis that was independent of ESRD cause. IOP change was predominantly associated with serum albumin levels and weight changes [2]. Yang and Jung reported that the mean IOP decreased significantly after hemodialysis in chronic renal failure patients [3,31]. The proposed mechanism of IOP change was rarely reported. Tokuyama et al. reported that IOP reduction was related to ultrafiltration rate by the mean of albumin level. The increasing oncotic pressure caused by ultrafiltration leads to a decline in IOP at the end of dialysis. IOP change is greater with a higher plasma albumin level and degree of ultrafiltration [32]. While the other study was proposed by Broekema, who suggested that despite a decrease in blood osmotic pressure, other regulatory forces prevent significant fluctuations in IOP during the hemodialysis [33].

In conclusion, our study found that hemodialysis can affect various ocular parameters, specifically SFCT, RAC and RVC, which changed significantly following the hemodialysis. The changes in SBP, DBP, body weight following hemodialysis may be a consequence of systemic compensatory mechanisms. Additionally, large-scale randomized studies are needed to determine the effect of hemodialysis on SFCT, retinal vasodilatation, the relationship between SFCT, RAC, RVC and systemic parameters. Although BCVA, IOP and CMT did not change post-hemodialysis in ESRD patients in this study, the ongoing study is needed to further determine the controversial result.

## Data Availability

The datasets analyzed during the current study are available from the corresponding author on reasonable request.

## Abbreviations

BCVA: Best-corrected visual acuity
IOP: intraocular pressure
CMT: central macular thickness
SFCT: subfoveal choroidal thickness
RAC: retinal arteriolar caliber
RVC: retinal venular caliber
ESRD: end-stage renal disease
HD: hemodialysis
SBP: Systolic blood pressure
DBP: diastolic blood pressure
HDLC: high density lipoprotein cholesterol
LDLC: low density lipoprotein cholesterol
VLDLC: very low density lipoprotein cholesterol
HbA1c: glycosylated hemoglobin
